# Clinical Significance of Extracellular Volume of Myocardium (ECV) Assessed by Computed Tomography: A Systematic Review and Meta-Analysis

**DOI:** 10.3390/jcm14062066

**Published:** 2025-03-18

**Authors:** Adrian Martuszewski, Patrycja Paluszkiewicz, Rafał Poręba, Paweł Gać

**Affiliations:** 1 Department of Environmental Health, Occupational Medicine and Epidemiology, Wroclaw Medical University, Mikulicza-Radeckiego 7, 50-345 Wrocław, Poland; 2 Department of Neurology, Specialist Hospital in Walbrzych, 58-309 Wałbrzych, Poland; 3 Department of Emergency Medical Service, Wroclaw Medical University, Bartla 5, 50-367 Wrocław, Poland; 4 Department of Biological Principles of Physical Activity, Wroclaw University of Health and Sport Sciences, 51-612 Wroclaw, Poland; 5 Centre of Diagnostic Imaging, 4th Military Hospital, Weigla 5, 50-981 Wrocław, Poland

**Keywords:** myocardium, cardiac fibrosis, computed tomography, extracellular volume, cardiac imaging

## Abstract

**Background/Objectives**: Extracellular volume (ECV) of the myocardium, a biomarker of interstitial space and fibrosis, plays a critical role in cardiac disease diagnosis and prognosis. Although cardiac magnetic resonance imaging (MRI) is the gold standard for ECV assessment, computed tomography (CT) offers a viable alternative, particularly in patients with contraindications to MRI. This study aimed to assess whether CT-derived ECV is systematically elevated in cardiac diseases associated with myocardial fibrosis. **Methods**: A systematic search of PubMed and Web of Science up to January 2023 identified 364 studies, including 16 from registers and 4 from manual searches. After exclusions, 73 studies were included in the systematic review. Of these, 15 provided quantitative data on group sizes, mean ECV values, standard deviations, and imaging modalities (CTA, DECT, LIE-DECT) and were analyzed in the meta-analysis. Standardized mean differences (SMD) were calculated using *Cochrane Handbook* formulas. Statistical analyses employed random-effects models (R version 4.4.2). **Results**: The pooled analysis showed that ECV was significantly higher in pathological groups compared to controls (SMD 1.60; 95% CI: 1.23–1.96; I^2^ = 84.6%). Elevated ECV correlated with worse clinical outcomes, including higher mortality in heart failure and advanced myocardial fibrosis in amyloidosis and cardiomyopathies. Subgroup analyses demonstrated that advanced CT techniques (DECT, LIE-DECT) and CTA provided comparable diagnostic accuracy. **Conclusions**: CT-derived ECV is a reliable, non-invasive marker of myocardial fibrosis, offering diagnostic and prognostic value similar to MRI. Standardizing CT protocols and conducting multicenter studies are essential to validate its broader clinical application.

## 1. Introduction

Cardiovascular diseases are the leading cause of death in Europe [1]. Extracellular volume (ECV) is a key cardiac imaging parameter that provides insight into myocardial pathologies such as fibrosis and inflammation. The extracellular matrix serves as a connective tissue scaffold for myocardial cells, where collagen fibers are synthesized and degraded. Dysregulation of this process, driven by inflammation, ischemia, oxidative stress, or neurohormonal activation, results in myocardial fibrosis. ECV is also recognized as a predictor of cardiovascular events [2].

Computed tomography (CT) enables ECV measurement with accuracy comparable to magnetic resonance imaging (MRI) [3]. This is particularly beneficial for patients in whom MRI, the gold standard, is contraindicated or unavailable [4]. Advances in CT technology, such as high-resolution imaging and improved algorithms, have enhanced myocardial ECV quantification. CT-derived ECV indirectly assesses structural myocardial changes linked to fibrosis, inflammation, and fluid shifts, offering valuable clinical insights without directly measuring specific pathologies.

ECV also aids in detecting diffuse myocardial fibrosis, a hallmark of conditions like hypertrophic and dilated cardiomyopathies and post-infarction remodeling. ECV alterations detected by CT may reveal subtle myocardial changes not always evident with late gadolinium enhancement (LGE) MRI, particularly in cases of diffuse fibrosis. Furthermore, ECV correlates with patient age, highlighting its role in distinguishing physiological from pathological cardiac remodeling [5].

This review evaluates the clinical significance of CT-derived myocardial ECV in cardiac diseases by critically analyzing methodologies, results, and clinical implications. This systematic review and meta-analysis was designed to determine whether CT-derived ECV is consistently elevated across diverse cardiac conditions associated with myocardial fibrosis and to examine its relationship with clinical outcomes, as well as to evaluate the consistency of ECV measurements across different imaging modalities.

### 1.1. Myocardial ECV: Morphology and Pathology

Myocardial flexibility (i.e., myocardial compliance or elasticity), defined as the ability of the heart to stretch and contract, is a key determinant of cardiac function. Impaired myocardial flexibility is associated with heart failure and various cardiovascular diseases, where structural changes in the extracellular matrix contribute to pathological remodeling. ECV quantification provides an indirect measure of interstitial space expansion due to fibrosis, inflammation, or infiltration. However, while ECV measurement reflects myocardial remodeling, it does not directly differentiate between fibrosis and inflammation.

Diffuse myocardial fibrosis, characterized by excessive collagen deposition in the interstitial space, is a major factor contributing to elevated ECV. Fibrotic processes can be classified as interstitial fibrosis, infiltrative fibrosis, and replacement fibrosis, each with distinct pathological implications. Neurohormonal activation plays a key role in fibrosis development, stimulating fibroblast proliferation and excessive collagen synthesis. In cases of replacement fibrosis, triggered by cardiomyocyte necrosis, myocardial architecture is further disrupted, leading to increased ECV and impaired cardiac function. Adverse post-infarction remodeling is one of the mechanisms associated with ECV elevation, as increased ECV in remote myocardium has been shown to correlate inversely with left ventricular ejection fraction (LVEF) [5]. Aging also contributes to progressive myocardial fibrosis. Histological studies indicate an age-related increase in collagen deposition, which has been linked to overexpression of TGF-β1 and reduced activity of matrix metalloproteinases (MMPs), leading to extracellular matrix remodeling, increased myocardial stiffness, and diastolic dysfunction [6,7].

Inflammation promotes pathological ECV expansion by enhancing vascular permeability, allowing fluids, proteins, and immune cells to infiltrate the interstitial space. Pro-inflammatory cytokines, including IL-1, IL-6, and TNF, disrupt the balance of collagen synthesis and degradation, driving extracellular matrix remodeling, myocardial stiffness, and diastolic dysfunction [7,8]. Advanced imaging techniques, particularly ECV quantification, effectively detect both overt fibrosis and subclinical abnormalities. ECV measurements precisely differentiate infarcted, fibrotic, and normal myocardium [5].

In infiltrative cardiomyopathies, such as amyloidosis and sarcoidosis, ECV expansion results from extracellular protein accumulation or granulomatous inflammation. In cardiac amyloidosis, misfolded amyloid proteins accumulate within the extracellular matrix, increasing interstitial space volume and impairing myocardial function. Treibel et al. [9] reported significantly higher ECV in transthyretin amyloidosis (ATTR) compared to light-chain amyloidosis (AL) (0.56 ± 0.11 vs. 0.43 ± 0.12, *p* < 0.05). Similarly, in sarcoidosis, granuloma formation and chronic inflammation disrupt myocardial integrity, further elevating ECV [8,10].

**Figure 1:**
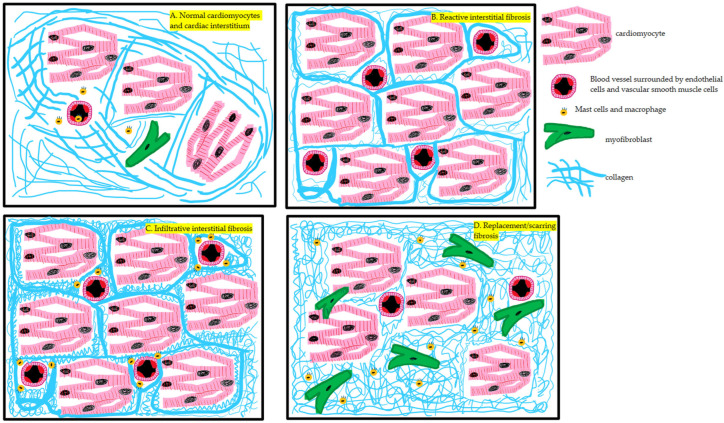
Structural and histological changes associated with myocardial fibrosis. (**A**) Normal myocardium, characterized by cardiomyocytes surrounded by minimal extracellular matrix and sparse collagen fibers. (**B**) Reactive interstitial fibrosis with increased interstitial collagen deposition, typically seen in hypertension or diabetes. Note the absence of inflammatory cell infiltration. (**C**) Infiltrative fibrosis showing marked extracellular accumulation, exemplified by amyloid deposits accompanied by mast cells and macrophages, disrupting normal myocardial architecture. (**D**) Replacement fibrosis resulting from cardiomyocyte necrosis, characterized by dense collagen scarring, increased myofibroblast presence, and loss of myocardial tissue, commonly observed after myocardial infarction.

**Figure 2:**
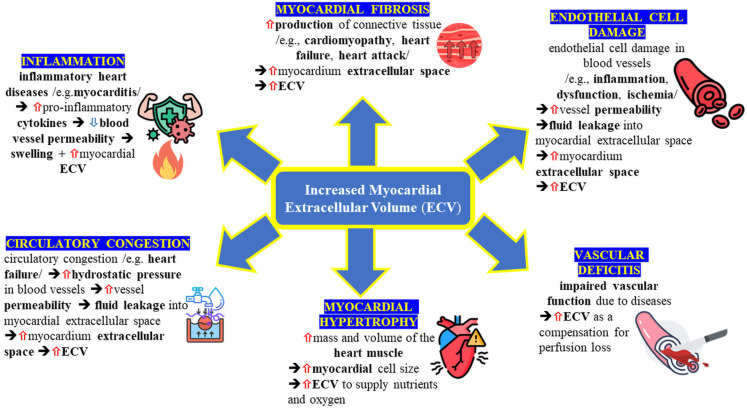
Mechanisms contributing to increased myocardial extracellular volume (ECV). This schematic summarizes various pathological processes that can lead to myocardial ECV expansion, including myocardial fibrosis (due to increased connective tissue production in conditions like cardiomyopathy, heart failure, or myocardial infarction), endothelial cell damage (inflammation or ischemia causing increased vascular permeability), vascular deficits (impaired perfusion leading to compensatory ECV increase), myocardial hypertrophy (increased cell size and mass demanding higher extracellular support), circulatory congestion (elevated hydrostatic pressure and fluid leakage into the myocardium, as seen in heart failure), and inflammation (cytokine-driven increased vessel permeability causing myocardial edema). These processes may act individually or collectively, resulting in elevated myocardial ECV and subsequent impairment of cardiac function. Red arrows (⇧) indicate an increase or elevation of the respective parameter and blue arrows (⇩) indicate a decrease or reduction in the corresponding parameter. Icons in this figure were obtained from Flaticon.com, designed by: Freepik, Futuer, kliwir art, Vectors Market, IconBaandar, Eucalyp, Sir.Vector, iconixar, Infinite Dendrogram.

Not all increases in ECV indicate pathology. For example, physiological hypertrophy in athlete’s heart often occurs without significant ECV elevation, whereas in hypertrophic cardiomyopathy (HCM), elevated ECV reflects fibrosis and structural disorganization [8,10]. Differentiating between physiological and pathological ECV expansion is crucial for accurate diagnosis and risk stratification. Non-invasive quantification of ECV using advanced CT imaging enables the detection of early myocardial remodeling, disease progression, and cardiovascular risk stratification [6,7,10]. Figure 1 illustrates structural changes associated with myocardial fibrosis and inflammation, while Figure 2 summarizes key mechanisms leading to ECV elevation, including inflammation, fibrosis, and infiltrative disease. Accurate assessment of ECV relies on robust imaging modalities, primarily MRI and CT.

### 1.2. Assessing ECV

Key imaging modalities for ECV assessment include MRI and CT. MRI is the gold standard for ECV quantification due to its high precision in tissue characterization and superior soft tissue contrast. Compared to CT, it offers greater sensitivity in detecting diffuse myocardial fibrosis while avoiding ionizing radiation, making it suitable for longitudinal studies. ECV is calculated using T1 mapping performed before and after contrast administration, combining myocardial and blood T1 relaxation times with hematocrit levels [4]. The following formula incorporates the relaxation rates of the myocardium (‘R1_myocardium_’) and blood (‘R1_blood_’) alongside hematocrit:

$$ECV=\frac{{R1}_{myocardium}}{{R1}_{blood}}\times \left(1-hematocrit\right)$$

CT is widely used due to its availability and rapid acquisition. ECV is calculated from pre- and post-contrast images by comparing Hounsfield unit (HU) values of the myocardium and blood [11].

ECV with CT is calculated using the difference in Hounsfield units (HU) between pre-contrast and post-contrast images of the myocardium (ΔHU_LVm_) and blood (ΔHU_b_), adjusted for hematocrit, as shown in the following equation:

$$ECV=\left(1-hematocrit\right)\times \frac{{∆HU}_{LVm}}{{∆HU}_{b}}$$

Measurements are typically acquired 5–7 min after contrast administration, based on established protocols. CT offers high spatial resolution for anatomical assessment; however, ionizing radiation and contrast use limit repeatability, especially in patients with renal dysfunction.

**Figure 3:**
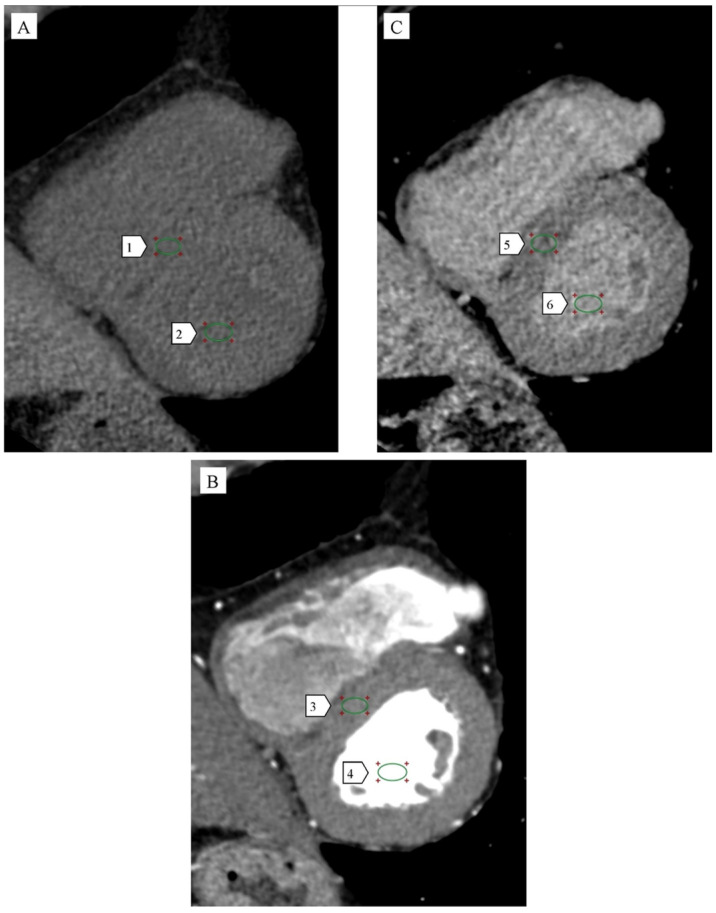
Cardiac computed tomography (CT). Attenuation method. Multiplanar reconstruction (MPR). Short axis. Level of middle segments of the left ventricle. (**A**) Native phase. **1.** HU myocardium in the native phase: 43.83 HU. **2.** HU LV blood in the native phase: 47.32 HU. (**B**) Angiographic phase CT (CCTA). **3.** HU myocardium in CCTA: 112.31 HU. **4.** HU LV blood in CCTA: 594.81 HU. (**C**) Delayed phase (CT-LE). **5.** HU of myocardium in CT-LE: 78.07 HU. **6.** HU LV blood in CT-LE: 111.73 HU. Hematocrit: 0.46. ECV: 28.7%. This figure represents original material created by the authors.

Both MRI and CT estimate ECV by incorporating hematocrit levels and a ratio of imaging parameters (relaxation rates in MRI, attenuation in CT). However, unlike MRI, CT-based ECV is affected by contrast administration variability, patient circulation, and acquisition timing, which may impact measurement consistency. Figure 3 illustrates a CT-based protocol for ECV quantification, emphasizing multiphase acquisition. ECV was calculated using HU measurements from the native, angiographic, and delayed enhancement phases, integrated with the patient’s hematocrit. While CT-derived ECV can detect extracellular volume expansion, its sensitivity for subtle changes may be lower than MRI due to these dependencies.

Spectral imaging techniques, such as DECT and LIE-DECT, aim to improve ECV quantification by reducing contrast-related variability and better differentiating iodine distribution from myocardial tissue characteristics. These methods may enhance reproducibility in CT-based ECV assessment. These methods improve differentiation of iodine contrast distribution and myocardial composition, facilitating the detection of ECV alterations. Their application in assessing myocardial fibrosis, infiltrative cardiomyopathies, and ischemic injury offers advantages such as reduced artifact interference and enhanced image quality. This attenuation-based approach highlights the potential of CT-derived ECV for myocardial tissue characterization, though it remains influenced by contrast administration protocols and patient-specific hemodynamics.

Emerging methods, including positron emission tomography, assess myocardial fibrosis using targeted tracers but remain primarily research-oriented [12].

## 2. Materials and Methods

**Figure 4:**
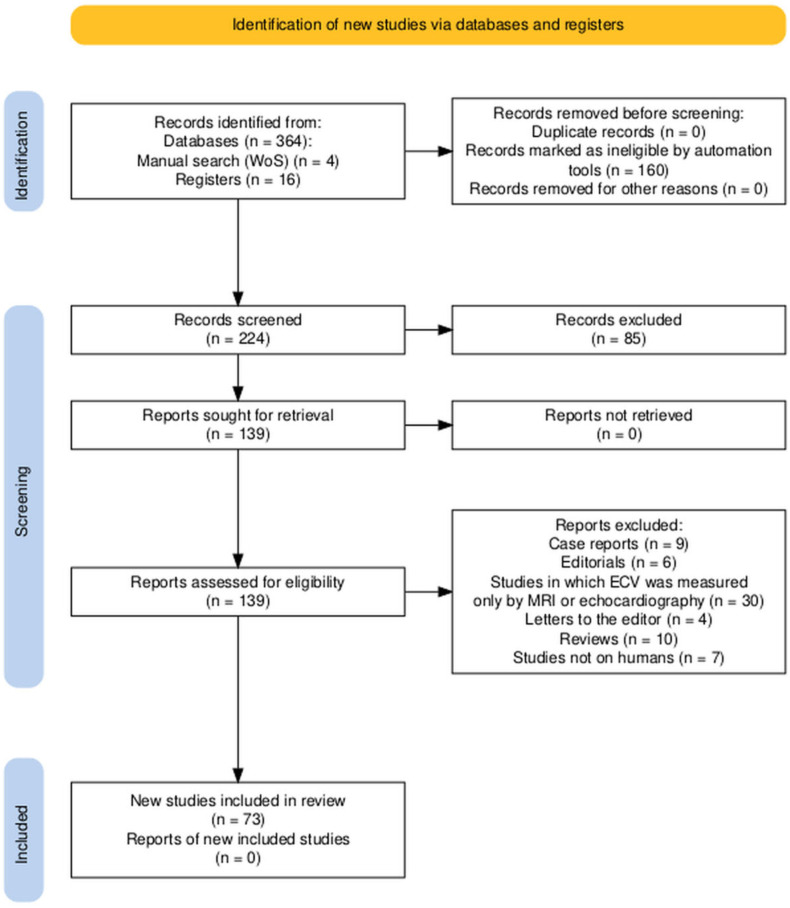
PRISMA flow diagram illustrating the study selection process [13]. Records were identified from databases (n = 364), registers (ClinicalTrials.gov, n = 16), and manual searches (Web of Science, n = 4). Of the 384 total records, 160 were excluded automatically based on predefined filters (English, MEDLINE, exclude preprints). The remaining 224 records were screened at the title and abstract level, with 85 records excluded during this stage. Following full-text assessment of 139 reports, 73 studies were included in the systematic review.

This systematic review followed PRISMA guidelines [13], ensuring transparency and replicability in study identification, screening, eligibility assessment, and inclusion. The PRISMA flow diagram (Figure 4) outlines each step of the selection process, providing a clear summary of study inclusion. The creation of this publication adhered to PRISMA guidelines and registration information (INPLASY202520109).

ClinicalTrials.gov was searched (10 June 2024) using the following terms: ((“heart”) OR (“cardiac”) OR (myocard)) AND ((“extracellular volume”) OR (“extracellular matrix”) OR (“ECV”))* under “Other Terms” and ((CT) OR (“computed tomography”) OR (CCTA) OR (coroCT)) under “Intervention/Treatment”. The review included only fully published, peer-reviewed studies, excluding unpublished trials to ensure methodological consistency and robust data. A total of 16 ClinicalTrials.gov studies assessed myocardial ECV using CT (Table 1).

PubMed was searched (7 January 2023) with the syntax ‘((“heart”) OR (“cardiac”) OR (myocard*)) AND ((“extracellular volume”) OR (“extracellular matrix”) OR (“ECV”)) AND ((CT) OR (“computed tomography”) OR (CCTA) OR (coroCT))’. Criteria included the following: (1) studies published within the last 5 years, (2) automated filters for: ‘English, MEDLINE, exclude preprints’, and (3) exclusion of case reports, editorials, letters to the editor, and studies measuring ECV only via MRI. Automated filters excluded ineligible records (n = 160), reducing irrelevant results. To address potential bias from automation, all remaining records (n = 224) were manually screened by two reviewers (AM and PP). Titles and abstracts were evaluated based on predefined criteria, excluding irrelevant studies such as: non-human studies, case reports, editorials, letters, reviews, preprints, or studies measuring ECV exclusively via MRI. Discrepancies were resolved through discussion, with a third reviewer consulted as needed. Only English-language studies were considered eligible to ensure consistency and methodological rigor. Any discrepancies during data abstraction or eligibility assessment were resolved through consensus discussions among reviewers, with consultation of a third reviewer when necessary to reach agreement. Full-text articles were reviewed to confirm eligibility. Manual searches of reference lists from retrieved articles and reviews in Web of Science identified four additional studies meeting inclusion criteria. A total of 85 publications were excluded after title and abstract screening. Following double-checks, 57 publications were included in the systematic review (53 from PubMed, 4 from manual searches), as depicted in Figure 4.

Study quality was assessed using tools tailored to specific designs. Diagnostic studies were evaluated with QUADAS-2 [14], focusing on patient selection, index tests, reference standards, and timing. Cohort studies were assessed using the Newcastle–Ottawa Scale (NOS) [15], which evaluates participant selection, group comparability, and outcome assessment. Systematic reviews were assessed with AMSTAR-2 [16], covering 16 domains, including protocol registration and risk of bias assessment. Non-randomized studies were evaluated with ROBINS-I [17], identifying moderate risk of bias in confounding and participant selection, with low risk across other domains. 

Following PRISMA guidelines, 15 studies were included in the meta-analysis. Eligibility criteria required studies to report quantitative ECV measurements and data sufficient for calculating standardized mean differences (SMD) and standard errors (SE) or 95%CI. Studies lacking quantitative ECV data or methodological detail were excluded. Group 1 represented the pathological group with higher ECV values, while group 2 served as the comparator group with lower values, ensuring consistent group definitions and minimizing discrepancies in data synthesis.

Effect sizes, including SMD, 95%CI, SE, and precision (1/SE), were calculated manually using formulas from the *Cochrane Handbook* [18]. For unequal group sizes (n_1_ ≠ n_2_), SD_pooled_ was calculated using a weighted formula based on group variances and sizes. For equal group sizes (n_1_ = n_2_), a simplified formula was applied, with N representing the total number of participants (N = n_1_ + n_2_). 95%CI were calculated using a critical value (Z = 1.96) for a two-tailed analysis.

$${SD}_{if\;not\;available}=SE\times \sqrt{n}$$

$${SD}_{pooled}=\sqrt{\frac{\left(n_{1}-1\right)\times {SD}_{1}^{2}+\left(n_{2}-1\right)\times {SD}_{2}^{2}}{n_{1}+n_{2}-2}}$$

$${SD}_{pooled}=\sqrt{\frac{{SD}_{1}^{2}+{SD}_{2}^{2}}{2}}$$

$$SMD=\frac{{mean}_{1}-{mean}_{2}}{{SD}_{pooled}}$$

$$SE=\sqrt{\frac{1}{N}+\frac{{SMD}^{2}}{2N}}$$

$$SE=\sqrt{\frac{1}{n_{1}}+\frac{1}{n_{2}}+\frac{{SMD}^{2}}{2\times \left(n_{1}+n_{2}\right)}}$$

95%CI=SMD±Z×SE

Statistical analyses were performed using R (version 4.4.2 “Pile of Leaves”) within RStudio (2024.12.0 Build 467), utilizing the meta and metafor packages. A random-effects model was employed to pool SMDs, with heterogeneity assessed through the I^2^ statistic, τ^2^ variance, and Cochran’s Q. The random-effects model was selected due to anticipated methodological and clinical heterogeneity across included studies, such as variations in patient populations, imaging modalities, and measurement protocols. Given the observed high heterogeneity (I^2^ > 75%), this model allows for more conservative and realistic estimation of pooled effects compared to a fixed-effects model, which assumes homogeneity among included studies. Subgroup analyses examined differences between imaging modalities (CTA vs. DECT/LIE-DECT), while leave-one-out sensitivity analyses evaluated the influence of individual studies on the pooled results. Funnel plot asymmetry was analyzed using Egger’s test to assess potential publication bias. Meta-regression analyses were conducted using the metafor package in R. The functions “rma()” and “metagen” were applied after preparing the dataset and incorporating potential moderating variables.

This systematic review and meta-analysis included the following patients (P): amyloidosis, aortic stenosis (AS), heart failure with preserved ejection fraction (HFpEF), dilated cardiomyopathy (DCM), myocarditis, and those undergoing atrial fibrillation ablation or cardiotoxic treatments. Studies were eligible if they reported quantitative ECV measurements obtained via CT. Extracted data included sample sizes (n), mean ECV values, standard deviations (SD), and the CT technique used. The intervention (I): ECV assessment using standard CTA and advanced imaging techniques, such as dual-energy CT (DECT) and late iodine enhancement DECT (LIE-DECT). Comparisons (C): between study groups (group 1 vs. group 2) and across imaging modalities (CTA vs. DECT/LIE-DECT). The outcome (O): SMD in ECV values between groups, reported with 95%CI, SE, and precision (1/SE).

## 3. Results

Out of 384 records identified (364 from databases, 16 from registers, and 4 from manual search), 73 met the inclusion criteria. The selected studies examined a variety of applications of CT-derived ECV in cardiac conditions, including amyloidosis, cardiomyopathies, heart failure, and ischemic heart disease. Most studies were conducted in single centers.

**Figure 5:**
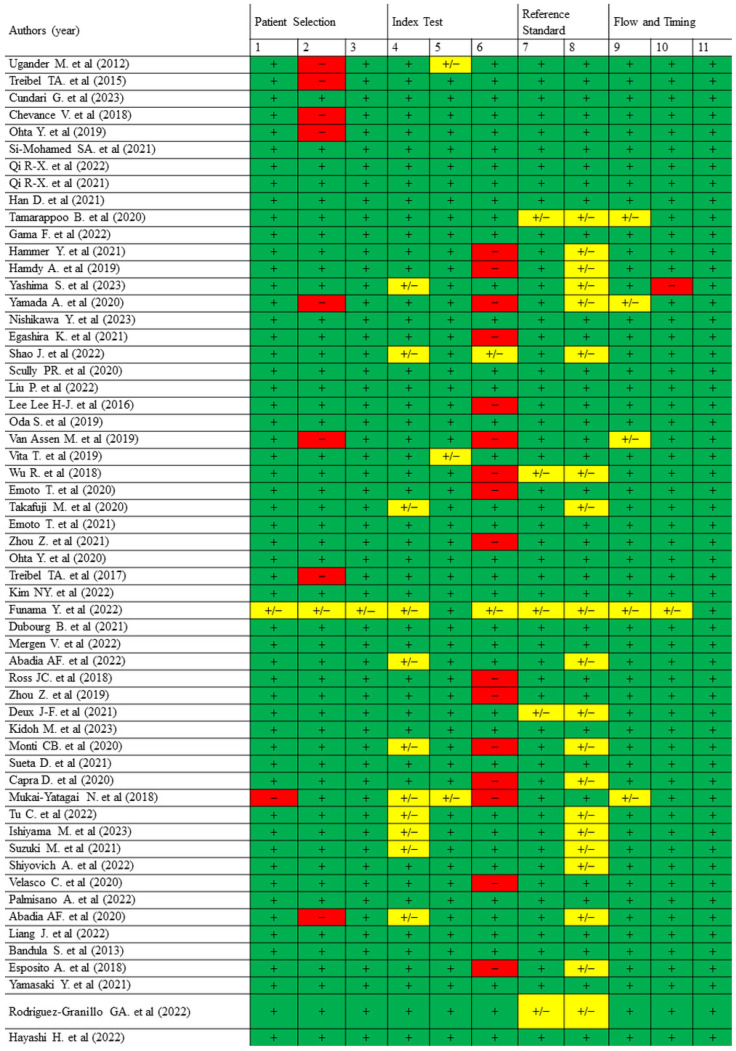
Quality assessment of diagnostic studies using the QUADAS-2 tool. This figure presents the quality assessment of diagnostic studies included in the systematic review, focusing on four key domains: patient selection, index test, reference standard, and flow and timing. Results are categorized as “+” (green), “−” (red), or “+/−” (yellow) to indicate whether the criteria were fully met, not met, or partially met, respectively. Abbreviations: +, yes; −, no; +/−, unclear. Signaling questions: (**1**) Were patients consecutively or randomly selected?; (**2**) Was a case-control design avoided?; (**3**) Were exclusions justified?; (**4**) Were results interpreted without knowledge of the reference standard?; (**5**) Were CT protocols standardized?; (**6**) Were diagnostic thresholds pre-specified?; (**7**) Did the reference standard correctly classify the condition?; (**8**) Was the interpretation of the reference standard blinded?; (**9**) Was the time interval between tests appropriate?; (**10**) Did all patients undergo the same tests?; (**11**) Were all patients accounted for in the analysis? [5,9,11,19,20,21,22,23,24,25,26,27,28,29,30,31,32,33,34,35,36,37,38,39,40,41,42,43,44,45,46,47,48,49,50,51,52,53,54,55,56,57,58,59,60,61,62,63,64,65,66,67,68,69,70,71,72].

**Figure 6:**
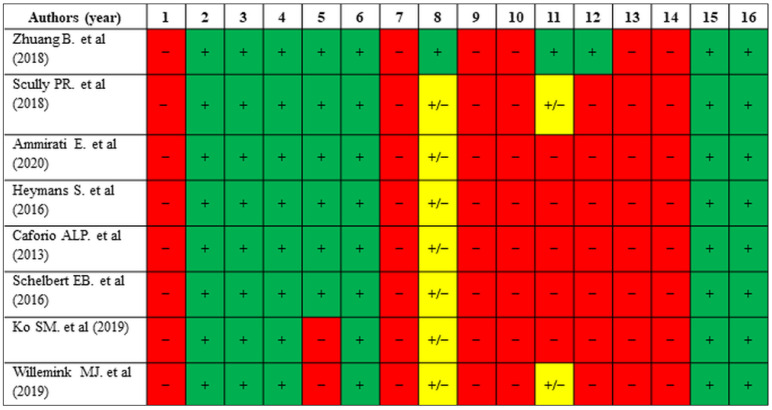
Quality assessment of systematic reviews using the AMSTAR-2 tool. This figure summarizes the quality assessment results for systematic reviews included in this study, evaluating 16 domains such as protocol registration, study selection, risk of bias assessment, and the validity of conclusions. Results are categorized as “+” (green), “−” (red), or “+/−” (yellow) to denote full compliance, non-compliance, or partial compliance with each criterion. Abbreviations: +, yes; −, no; +/−, partially yes. Domain questions: (**1**) Protocol Registration—Was a protocol registered before the review was conducted?; (**2**) Review Question—Are the research question and inclusion criteria clear and appropriate?; (**3**) Study Selection—Was the selection of studies explained and justified?; (**4**) Literature Search—Was the literature search comprehensive and transparent?; (**5**) Duplicate Screening—Were studies screened and data extracted in duplicate?; (**6**) Study Characteristics—Were characteristics of the included studies provided?; (**7**) Risk of Bias Assessment—Was the risk of bias in individual studies assessed adequately?; (**8**) Statistical Methods—Were the methods for meta-analysis (if performed) appropriate?; (**9**) Funding Sources—Were funding sources of the included studies reported?; (**10**) Risk of Bias in Interpretation—Was the risk of bias considered when discussing findings?; (**11**) Heterogeneity—Was heterogeneity explored and adequately addressed?; (**12**) Publication Bias—Was the likelihood of publication bias assessed and discussed?; (**13**) Conflicts of Interest—Were conflicts of interest of the reviewers and the included studies disclosed?; (**14**) Adequacy of Search Updates—Was the search strategy updated during the review process (if applicable)?; (**15**) Data Integrity—Were efforts made to ensure accuracy in data collection and reporting?; (**16**) Conclusions—Were the conclusions justified by the evidence presented? [2,3,4,6,7,10,74,75].

The evaluation with QUADAS-2 (Figure 5) indicated strong methodological quality across diagnostic studies, although concerns were identified in the “Index Test” and “Flow and Timing” domains in certain publications. Cohort studies assessed using the NOS (Table 2) demonstrated robust methodological quality, particularly in participant selection and outcome measurement, but comparability across studies varied. In NOS, high scores were achieved for selection and comparability, but minor limitations were identified in outcome measurement. Non-randomized interventional studies evaluated with ROBINS-I (Table 3) were generally classified as having moderate risk of bias, with the greatest concerns arising in the domains of confounding and participant selection. Systematic reviews assessed with AMSTAR-2 (Figure 6) showed consistent strengths in study selection and bias assessment but frequently lacked protocol registration and publication bias evaluation.

**Table 2:** Quality assessment of cohort studies using the Newcastle–Ottawa Scale (NOS). This table provides a detailed assessment of cohort study [8] using the NOS framework, which evaluates three key domains: selection of participants, comparability of groups, and assessment of outcomes. Results are presented using a star-based system, with each star indicating that a specific criterion has been met.

Authors (Year)	Selection				Comparability	Outcome		
	1	2	3	4	5	6	7	8
Frustaci A. et al. (2007)	*	*	*	*	**	*	*	*

Abbreviations: *—star. Signaling question (possible response): (1) Was the cohort representative of the population? (*/0); (2) Was the non-exposed cohort appropriately selected? (*/0); (3) Was exposure objectively ascertained? (*/0); (4) Was the cohort free of the outcome at the start of the study? (*/0); (5) Were confounding factors controlled (1 star for each factor)? (**/*/0); (6) Was the outcome assessed objectively/blinded? (*/0); (7) Was the follow-up duration sufficient? (*/0); (8) Were follow-up data complete? (*/0).

**Table 3:** Risk of bias assessment for non-randomized studies using the ROBINS-I tool. This table presents the quality assessment results for non-randomized study [73] included in the review, evaluated with the ROBINS-I tool. Seven domains were analyzed, including confounding factors, participant selection, intervention classification, and handling of missing data. Each domain can be rated as low risk, moderate risk, serious risk, critical risk.

Authors (Year)	Confounding	Selection Bias	Classification Bias	Deviation from Intervention	Missing Data	Outcome Measurement Bias	Reporting Bias	Overall Bias
Egashira et al. (2022)	M	M	L	L	L	L	L	M

Abbreviations: L, low risk; M, moderate risk.

A total of 15 studies were included in the meta-analysis, encompassing 1129 participants. The studies employed three main imaging modalities for ECV quantification: DECT, LIE-DECT, and standard CTA. The clinical groups varied widely, including conditions such as amyloidosis, HFpEF, DCM, myocarditis, and post-anthracycline cardiotoxicity (CTRCD, Cancer Therapeutics-Related Cardiac Dysfunction). Table 4 summarizes the study characteristics, imaging methods, sample sizes, mean ECV values, and calculated SMD.

**Figure 7:**
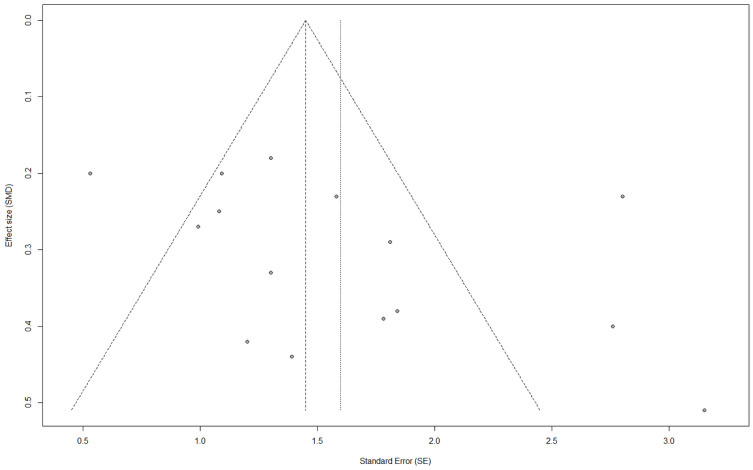
The funnel plot evaluates the presence of publication bias in the meta-analysis of ECV measurements. The X-axis represents the standardized mean difference (SMD), and the Y-axis denotes the standard error (SE). The plot is relatively symmetrical, suggesting no significant publication bias, although some minor asymmetry could indicate potential reporting bias. Each dot represents an individual study included in the meta-analysis.

The funnel plot (Figure 7) evaluates the presence of publication bias in the included studies. Visual inspection of the plot indicates a relatively symmetrical distribution, suggesting the absence of significant publication bias. This finding is supported by Egger’s test (t = 1.64, *p* = 0.1089), which did not reveal statistically significant asymmetry.

**Figure 8:**
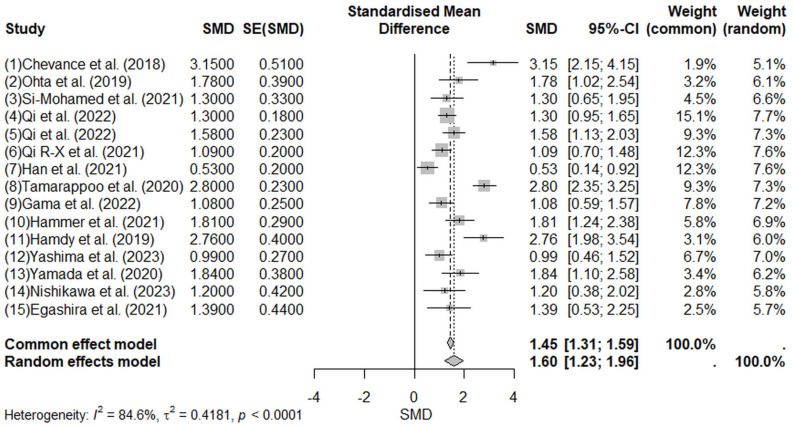
Forest plot illustrating the pooled standardized mean difference (SMD) for extracellular volume (ECV) measured by computed tomography (CT) in pathological versus control groups [19,20,21,22,23,24,25,26,27,28,29,30,31,32]. A positive SMD indicates higher ECV values associated with pathological conditions. The pooled SMD of 1.60 (95% CI: 1.23–1.96, random-effects model) demonstrates significantly higher ECV in pathological groups. Considerable heterogeneity (I^2^ = 84.6%) reflects methodological and clinical variability among included studies. Squares represent individual study estimates, horizontal lines indicate 95% confidence intervals, and the diamond represents the pooled effect size.

The pooled analysis shown in Figure 8 indicated significantly higher ECV values in pathological groups compared to controls, with a pooled SMD of 1.60 (95% CI: 1.23–1.96, random-effects model). The positive direction of the SMD indicates that increased ECV values are associated with pathology. Significant heterogeneity was observed among included studies (I^2^ = 84.6%; tau^2^ = 0.4181; Q = 90.69; *p* < 0.0001), suggesting considerable variability attributable primarily to differences in study populations, imaging protocols, and measurement methodologies.

**Figure 9:**
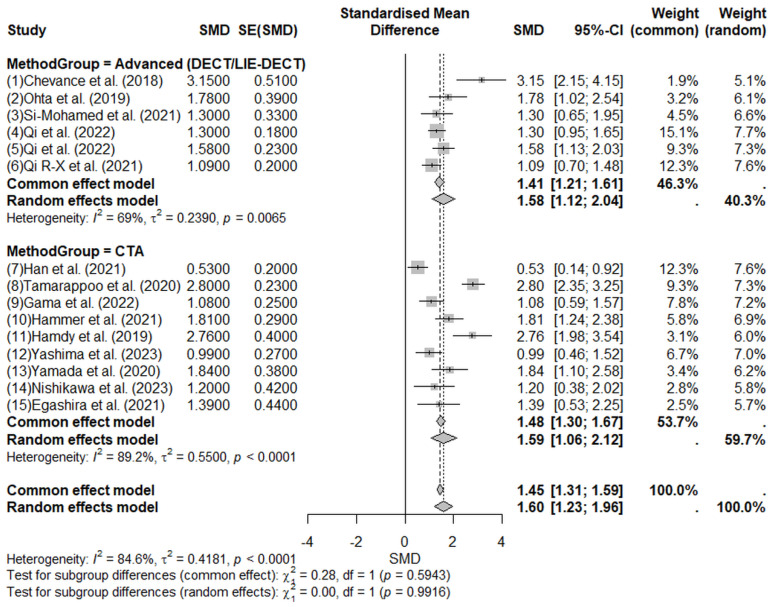
Subgroup forest plot stratified by imaging modality (advanced DECT/LIE-DECT vs. standard CTA), comparing extracellular volume (ECV) in pathological groups versus controls [19,20,21,22,23,24,25,26,27,28,29,30,31,32]. Positive SMDs across both subgroups indicate increased ECV values associated with pathological states. Advanced imaging yielded a pooled SMD of 1.58 (95% CI: 1.12–2.04; I^2^ = 69%), indicating moderate heterogeneity. Standard CTA showed a similar pooled SMD of 1.59 (95% CI: 1.06–2.12; I^2^ = 89.2%), reflecting higher variability. No statistically significant difference between subgroups was observed (*p* = 0.9916). Squares represent individual study estimates, horizontal lines indicate 95% confidence intervals, and the diamond represents the pooled effect size.

Subgroup analysis based on the imaging techniques used (Figure 9) revealed that studies utilizing advanced imaging methods (DECT and LIE-DECT; 6 studies, n = 465) [19,20,21,22,23] consistently demonstrated significantly higher ECV values in pathological groups compared to controls, with an SMD of 1.58 (95% CI: 1.12–2.04, random-effects model). Moderate, statistically significant heterogeneity was present within this subgroup (I^2^ = 69%; tau^2^ = 0.2390; *p* = 0.0065). Similarly, studies employing standard CTA imaging (9 studies, n = 664) [24,25,26,27,28,29,30,31,32] also showed significantly elevated ECV in pathological groups (SMD = 1.59; 95% CI: 1.06–2.12, random-effects model), though heterogeneity was notably higher (I^2^ = 89.2%; tau^2^ = 0.5500; *p* < 0.0001).

**Figure 10:**
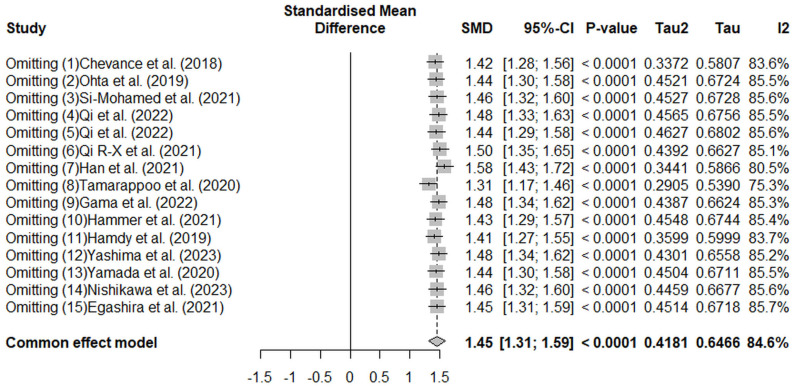
This sensitivity analysis examines the robustness of the pooled standardized mean difference (SMD) by sequentially excluding each study [19,20,21,22,23,24,25,26,27,28,29,30,31,32]. The results show stable pooled SMD estimates (range: 1.31–1.58), with no single study disproportionately influencing the overall effect size. Despite the high heterogeneity (I^2^ = 84.6%), the stability supports the reliability of the meta-analysis findings across different studies and methodologies. Squares represent individual study estimates, horizontal lines indicate 95% confidence intervals, and the diamond represents the pooled effect size.

The robustness of the pooled results was evaluated using sensitivity analysis (Figure 10). Sequential exclusion of individual studies did not substantially alter the pooled SMD, which ranged between 1.31 and 1.58. This consistency indicates that no single study had a disproportionate impact on the overall effect size. Despite high heterogeneity (I^2^ = 84.6%, *p* < 0.001), the stability of results underscores the reliability of the meta-analysis findings.

Due to high heterogeneity identified in the meta-analysis results (I^2^ = 84.6%), a meta-regression was conducted to identify potential moderating factors. The meta-regression revealed no significant impact of imaging methodology (CTA vs. DECT/LIE-DECT) on the SMD (QM = 0.0093, *p* = 0.9230). Imaging method as a moderator did not explain any portion of the observed heterogeneity (R^2^ = 0.00), and heterogeneity remained high following the meta-regression (I^2^ = 85.47%). Similarly, sample size was not found to significantly moderate the SMD (QM = 0.2134, *p* = 0.6441). Number of participants did not account for any portion of heterogeneity (R^2^ = 0.00), with heterogeneity remaining elevated after the analysis (I^2^ = 85.53%). We further examined whether the type of underlying disease influenced the SMD by categorizing studies into three groups: cardiomyopathies (including dilated cardiomyopathy, amyloidosis, HFpEF, myocarditis), ischemic conditions (such as myocardial infarction, post-ablation patients, aortic stenosis), and other diseases (dialysis patients, oncological diseases, etc.). Disease type did not significantly moderate the SMD (QM = 1.3895, *p* = 0.4992) and did not significantly reduce the heterogeneity (I^2^ = 85.18%). Additionally, a meta-regression analyzing the interaction between disease type and imaging method also showed no significant effect (*p* = 0.6881), with heterogeneity still remaining high (I^2^ = 85.44%).

## 4. Discussion

Heterogeneity in the meta-analysis reveals more pronounced methodological and population differences in CTA studies than in DECT studies, potentially limiting the generalizability of results. This variation likely arises from inconsistencies in imaging protocols, patient selection criteria, and scanner technologies. However, the high methodological quality of most studies supports the reliability of the review’s conclusions. Quality assessments using QUADAS-2, NOS, ROBINS-I, and AMSTAR-2 established a robust evidence base. Specifically, the QUADAS-2 evaluation indicated generally high methodological quality of diagnostic studies, though concerns were noted, particularly regarding standardization of CT protocols and timing between index and reference tests. The NOS assessment highlighted strengths in participant selection and outcome assessment for cohort studies, but comparability between groups was less consistently addressed. According to ROBINS-I, non-randomized studies primarily showed moderate risk of bias due to potential confounding and participant selection bias. Finally, systematic reviews evaluated by AMSTAR-2 consistently lacked protocol registration and publication bias assessment, which limits the confidence in their conclusions. These quality considerations emphasize caution in interpreting results, particularly when generalizing findings beyond the reviewed populations. By integrating advanced imaging techniques with rigorous methodology, the meta-analysis highlights the diagnostic and prognostic value of ECV in myocardial fibrosis, with particular emphasis on the superior consistency of DECT and LIE-DECT.

Due to high heterogeneity observed among the studies, a meta-regression was additionally performed; however, no significant moderators that could explain the variability of results were identified. The observed heterogeneity may be attributed to other unexamined factors, such as differences in study protocols, patient characteristics, or diagnostic criteria. This is also an important limitation of our analysis.

### 4.1. Methods of Obtaining ECV from CT

Our meta-analysis highlights the diagnostic and prognostic significance of CT-derived ECV across imaging modalities and clinical conditions, particularly in cardiac amyloidosis and HFpEF, where elevated ECV correlates with adverse outcomes. Advanced imaging techniques, such as DECT and LIE-DECT, demonstrated reduced heterogeneity (I^2^ = 69%) compared to standard CTA (I^2^ = 89.2%), reflecting their improved reliability in ECV quantification.

ECV assessments can be global, segmental, or remote, each offering unique insights into myocardial health. Global ECV evaluates overall myocardial health, segmental ECV focuses on specific regions, and remote ECV examines areas distant from myocardial damage. Comparisons between CT-derived ECVsub and ECViodine revealed superior reproducibility and interobserver agreement for ECViodine, with higher ICCs for global (0.804 vs. 0.607) and remote ECV (0.859 vs. 0.669) [33]. These findings align with meta-analysis results, emphasizing the superior specificity and reproducibility of DECT-derived measurements.

CT and MRI measure ECV non-invasively, eliminating the need for biopsy [3]. CT is advantageous for patients contraindicated for MRI. Myocardial iodine concentration (MIC) effectively differentiates cardiac amyloidosis from other cardiomyopathies, with 100% sensitivity and 92% specificity, correlating strongly with ECV (r = 0.92, *p* < 0.0001) [19]. CT-derived ECV closely matches MRI values (46.4% vs. 49.2%) [76] and can identify dual aortic stenosis and cardiac amyloidosis in TAVR patients, using simplified protocols that reduce radiation exposure by 75% (DLP = 90 mGy·cm; effective dose = 2.3 mSv) [34]. A 31% CT-ECV threshold distinguishes myocardial segments with MRI-defined LGE, with sensitivity and specificity of 83.1% and 93.3%, respectively [35]. However, these cut-off values require cautious interpretation, as they were derived from limited patient populations, and their direct translation into clinical practice might be restricted by variability in scanner types, acquisition parameters, and patient-specific factors. Prospective validation in diverse cohorts and standardized imaging protocols are essential to ensure clinical applicability.

DECT reliably measures ECV in non-ischemic cardiomyopathy (CMP) with minimal bias (0.06%) and high interobserver reliability (ICC = 0.987). Patients with hypertrophic CMP, dilated CMP, amyloidosis, or sarcoidosis show significantly elevated ECV compared to controls (*p* < 0.01) [36]. When MRI is unavailable, CT provides accurate ECV measurement [74]. DECT achieves MRI-comparable accuracy (r^2^ = 0.94, *p* < 0.005) and avoids misregistration issues inherent to SECT, which requires pre- and post-contrast imaging [37].

DECT avoids this issue using iodine density imaging. Both SECT and DECT effectively differentiate infarcted from healthy myocardium, with similar ECV values (33.4% vs. 34.9%, *p* >0.005) [38]. However, DECT demonstrates superior specificity for distinguishing normal myocardium from non-ischemic CMP (85.7% vs. 60%) [20].

MRI-derived ECV from 18 myocardial segments predicts MACE in dilated cardiomyopathy, with each ECV abnormality increasing risk by 18% (*p* = 0.0003) [39]. Advances in CT imaging, such as iterative reconstruction, improve ECV accuracy and radiation efficiency. Model-based iterative reconstruction outperforms hybrid methods by reducing beam-hardening artifacts [40,41,75]. DECT ensures stable ECV measurements despite reduced contrast doses [42], outperforming SECT in precision and radiation efficiency, particularly in doxorubicin-induced cardiomyopathy [43,44].

Synthetic ECV eliminates the need for blood sampling by estimating hematocrit from imaging. Techniques like Virtual Unenhanced attenuation (VUE) correlate non-contrast CT attenuation with blood density, achieving minimal bias and strong agreement with traditional methods [38,45,46,47].

High-resolution images obtained with a 320-row detector CT enhance ECV precision, though lower radiation doses increase variability. Differences in ECV values between scanned layers highlight the importance of protocol optimization. Dual-layer CT (DLCT) improves material differentiation and image quality through dual-energy analysis, outperforming traditional CT [48]. DLCT achieves strong correlations with MRI (r = 0.79–0.83), though intersegmental variability requires cautious interpretation [49]. Photon-counting detector CT (using iodine ratios with minimal Bland–Altman error) achieves high-resolution ECV quantification without pre-contrast scans, minimizing alignment errors [50].

Machine learning reduces ECV analysis time from 9.4 to 2.2 min using a five-step automated DECT-based algorithm, which includes (1) heart segmentation, (2) myocardial segmentation, (3) blood pool segmentation, (4) scan co-registration, and (5) ECV calculation. Automated methods demonstrate moderate agreement with manual techniques (ICC = 0.66, r = 0.68) and similar ECV values in cardiomyopathy patients (37.8% manual vs. 35.9% automated) and healthy controls (25.2% manual vs. 23.1% automated, *p* < 0.001). Both approaches effectively differentiate cardiomyopathy from healthy myocardium, with AUC values of 1 for manual and 0.97 for automated methods [51].

### 4.2. Amyloidosis

Amyloidosis, caused by amyloid accumulation in tissues, including the heart, leads to dysfunction and heart failure. One study [26] evaluated CT-derived ECV in systemic amyloidosis for cardiac remodeling and mortality assessment. In a cohort (49% ATTR, 51% AL), the mean ECV was 49%, with higher values in ATTR than AL (56% vs. 43%). Septal ECV correlated with left ventricular mass (r = 0.426), ejection fraction (r = 0.460), NT-proBNP (r = 0.563), and hsTnT (r = 0.546). ECV was identified as an independent mortality risk factor in ATTR (HR: 1.046, 95% CI: 1.003–1.090, *p* = 0.037). The myocardium-to-lumen ratio served as an additional marker for cardiac amyloid burden. CT-derived ECV correlated strongly with MRI, supporting CT as a viable alternative.

Further studies examined ECV for amyloidosis diagnosis. One investigation validated ECV’s role in confirming scintigraphy for amyloidosis detection [52]. Another study [53] in beagles demonstrated the reliability of contrast-enhanced CT-derived ECV for diffuse myocardial fibrosis, providing an option for MRI contraindications. A separate analysis [54] compared ECV and myocardial perfusion in cardiac amyloidosis (CA), non-amyloid hypertrophic cardiomyopathy (NACH), and normal myocardium. ECV was higher in CA (54.7%) than in NACH (34.6%) and normal myocardium (35.9%). A “baso-apical gradient” showed higher basal (57%) than apical ECV (52.5%), reflecting uneven amyloid deposition. ROC analysis indicated high diagnostic accuracy for basal ECV (AUC = 0.93, 95% CI: 0.89–0.98) and mean ECV (AUC = 0.92, 95% CI: 0.88–0.97). Elevated ECV (>56%) was associated with increased mortality risk (HR: 4.2, 95% CI: 1.4–11.8). Patients who died during follow-up (23 months) had higher ECV than survivors (57.7% vs. 53.8%, *p* < 0.05). ECV > 56% may guide treatment intensity and monitoring.

CT-ECV has also been explored as an MRI alternative. One study [55] assessed its ability to detect cardiac amyloidosis using ECV and the myocardium-to-lumen ratio, independent of non-contrast CT or hematocrit data. Patients with amyloidosis had higher ECV (52%) than those without (29%). Both ECV and the myocardium-to-lumen ratio independently predicted amyloidosis. At a 37% ECV cut-off, sensitivity and specificity were 90% and 92% (AUC = 0.97), respectively, while the myocardium-to-lumen ratio achieved 88% sensitivity and 92% specificity (AUC = 0.96). While promising, such cut-off thresholds should not be generalized without external validation, considering variability due to imaging techniques and patient characteristics. These findings highlight CT-ECV’s potential as a non-invasive MRI alternative, with the myocardium-to-lumen ratio offering a solution when hematocrit data are unavailable.

### 4.3. Oncology

Anthracycline-induced cardiotoxicity in breast cancer patients is associated with increased ECV. ECV measured by CT increased from 27% to 30% post-treatment, with the change being irreversible [56]. CT provides reliable, repeatable measurements, making it a valuable tool for monitoring cardiotoxicity and diagnosing CTRCD. CT-derived late myocardial enhancement and ECV results align with those from MRI, demonstrating its utility in cardio-oncology [57].

ECV values during anthracycline therapy were 26.2% in controls, 27.5% in patients without CTRCD, and 30.3% in those with CTRCD (*p* < 0.05), indicating its potential to identify at-risk patients [32]. In esophageal cancer patients undergoing radiotherapy, ECV increased from 27.9% pre-treatment to 30.3% shortly after and 31.4% at 420 days (*p* = 0.011). Post-treatment ECV correlated with the maximum radiotherapy dose (Spearman’s coefficient = 0.50, *p* < 0.05), suggesting it as a non-invasive marker of myocardial fibrosis [58,59].

In breast cancer patients, ECV significantly increased 12 months after anthracycline therapy (*p* < 0.01, Bonferroni analysis). Early increases in ECV correlated with lower baseline LVEF. ECV trends mirrored LVEF and global longitudinal strain (GLS), confirming its consistency [73].

Retrospective data further validated ECV as an early biomarker for cardiotoxicity, with increases from 26.76% pre-therapy to 31.32% at 61 days, 29.60% at 180 days, and 32.05% at 350 days. Higher ECV at later timepoints was observed in patients with cardiotoxicity (*p* < 0.001). ECV increases preceded LVEF reductions, making it a sensitive marker for early cardiotoxicity and a potential tool for timely interventions [60].

### 4.4. Transcatheter Aortic Valve Implantation and Aortic Stenosis

Severe AS is a life-threatening condition characterized by narrowing of the aortic valve, leading to restricted blood flow, chest pain, heart failure, and fainting. Transcatheter aortic valve implantation (TAVI), also known as transcatheter aortic valve replacement (TAVR), is a minimally invasive procedure for valve replacement, and ECV measurement via CT has become a valuable tool for predicting outcomes and identifying high-risk patients.

Higher preoperative ECV has been linked to worse outcomes after TAVI. Patients with high ECV (median 35%) had smaller reductions in left ventricular mass (LVM) post-TAVI, and fewer had reductions exceeding 20% compared to those with lower ECV (median 30%) [61].

High ECV was the strongest predictor of heart failure-related hospitalization (HR = 10.8; *p* < 0.05), with significantly worse outcomes shown by Kaplan–Meier analysis (*p* < 0.001). Pre-TAVI ECV also predicts LVEF recovery, with patients lacking improvement showing higher ECV (33.2% vs. 29.4%). Logistic regression indicated that each 1% ECV increase above 30% reduced the likelihood of recovery by 11%, underlining its prognostic value [24].

Higher ECV correlates with advanced heart failure symptoms, including worse NYHA class, larger left atrial volume, and lower LVEF, suggesting its utility in monitoring severe AS and predicting clinical outcomes [27]. DECT-derived ECV has been associated with worse clinical parameters such as higher BNP, poorer LVEF, and worse diastolic function (E/e′ ratio), as well as increased mortality and hospitalization risks, emphasizing its role in risk stratification and treatment planning. Correlation analysis showed associations between ECV and BNP (r = 0.395), E/e′ (r = 0.223), and LVEF (r = −0.251). High ECV was associated with higher risks of all-cause mortality and heart failure-related hospitalization. These findings highlight DECT-derived ECV as a valuable tool for risk stratification and personalized treatment planning in severe AS [62].

In patients with low-flow, low-gradient AS, ECV > 33% has been linked to poorer outcomes, including lower LVEF and a higher prevalence of atrial fibrillation, supporting its inclusion in preoperative evaluation [25]. An inverse relationship between serum albumin and ECV was observed, with lower albumin levels correlating with higher ECV and increased myocardial fibrosis (r = −0.7; *p* < 0.001) [63]. Monitoring albumin may offer a simple biomarker for identifying patients at higher risk of myocardial fibrosis in AS.

### 4.5. ECV in Myocarditis, Cardiomyopathy, and Other Cardiac Conditions

Myocarditis, an inflammatory condition of the heart muscle, may result from infections or autoimmune diseases. DECT was shown to effectively measure ECV for diagnosing acute myocarditis, with significantly higher ECV in affected patients compared to controls (34.18% vs. 30.04%; *p* < 0.001). An ECV threshold of 31.6% demonstrated 80% sensitivity and 78% specificity (AUC 0.835), positioning DECT as a cost-effective alternative to MRI [21]. Stable ECV values in non-infarcted myocardium up to 7 min post-contrast confirmed its reliability [28]. PET-CT with gadolinium contrast was used to assess ECV and myocardial blood flow, revealing increased ECV in infarcted myocardium, emphasizing ECV’s role as a biomarker for coronary artery disease [64]. A diagnostic protocol combining triple-rule-out CT and late-enhancement CT identified elevated ECV in myocarditis (median 30%), takotsubo cardiomyopathy, and cardiac amyloidosis, allowing comprehensive evaluation of acute chest pain in a single session [65]. DECT-derived ECV was proposed as a marker for cardiomyopathy, with 90.3% sensitivity and specificity at a cut-off of 29.5% [66]. In HFpEF, ECV correlated significantly with left ventricular mass (r = 0.32), left atrial volume (r = 0.35), septal thickness (r = 0.27), NT-proBNP (r = 0.63), and NYHA class (r = 0.31), highlighting its value in tracking disease progression [22]. ECV also predicted MACE in dilated cardiomyopathy. Patients with MACE had higher ECV (37.16% vs. 32.59%), with a threshold of 32.26% (AUC 0.74) showing 95% sensitivity and 51% specificity [29]. Spectral dual-layer CT correlated well with MRI-derived ECV (r = 0.79) in post-PCI patients, with excellent interobserver agreement (ICC = 0.85), supporting its use for myocardial assessment [67]. A DynEQ-CT study showed strong agreement with MRI-derived ECV in cardiac amyloidosis (r^2^ = 0.85, *p* < 0.001). ECV was significantly higher in amyloidosis than in severe aortic stenosis (0.54 ± 0.11 vs. 0.28 ± 0.04; *p* < 0.001), suggesting its utility in distinguishing hypertrophy causes [9]. Equilibrium contrast-enhanced CT correlated strongly with histological fibrosis (r = 0.71, *p* < 0.001) and MRI-derived ECV (r = 0.73), demonstrating its role in evaluating diffuse fibrosis, especially where MRI is unavailable. Practical advantages include shorter imaging times and workflow integration, though radiation exposure and image noise remain limitations [68].

### 4.6. Other Studies

ECV correlates positively with age (rho = 0.375) and end-diastolic volume (rho = 0.378) but inversely with ejection fraction (rho = −0.334). CT-based ECV assists in diagnosing troponin-positive chest pain without echocardiographic or electrocardiographic abnormalities, including myocarditis, subacute myocardial infarction, cardiac amyloidosis, or myocardial vasculitis [69,77].

In hemodialysis patients, ECV is significantly higher (33.8%) than in controls (26.6%) and correlates with the left atrial volume index (r = 0.54 in patients, r = 0.56 in controls), highlighting its role in tracking cardiovascular changes [30].

ECV in the right ventricle decreases after CTEPH treatment (26.1% vs. 29.1%) and correlates with pulmonary artery pressure (*p* < 0.01) and BNP (*p* < 0.05). This suggests its utility as a biomarker for disease severity and treatment response, with high measurement consistency supporting clinical use [70].

In heart failure risk assessment, ECV is higher in at-risk patients (31.3%) than in controls (27.1%) and correlates with NT-proBNP (r = 0.629) and left atrial volume, suggesting its utility as an early biomarker of myocardial changes [23].

Among ischemic stroke patients, ECV averaged 33.5%, but no differences were observed between stroke subtypes. Although ECV did not correlate with stroke ethology, late iodine enhancement and ECV changes provided insights into myocardial injury [71].

CT-ECV strongly correlates with MRI-ECV and associates with pulmonary artery pressure in PH patients. At the RV insertion point, CT-ECV acts as a biomarker for PH severity and offers a non-invasive alternative to MRI-ECV for assessing myocardial changes [72].

In AF patients undergoing catheter ablation, ECV predicts reverse remodeling (RR). Patients with RR had lower ECV (33% vs. 39%), and an ENL score (ECV, NT-proBNP, LVEDV) demonstrated high predictive accuracy (AUC 0.9583, sensitivity 92%, specificity 89%), aiding treatment planning [31].

In chronic coronary syndromes, CT plays a pivotal role as a comprehensive diagnostic tool, integrating both anatomical and functional assessment. ECV quantified via CT provides additional insights into myocardial structure and function, aiding in the diagnosis of coronary artery disease and cardiovascular risk stratification. Multimodal imaging in CCS is increasingly recognized, combining CCTA with perfusion and coronary flow reserve assessment techniques. Recent studies suggest that CT-ECV imaging, when integrated with methods such as FFR-CT, can improve patient risk classification and better identify those who may benefit from revascularization [78].

**Figure 11:**
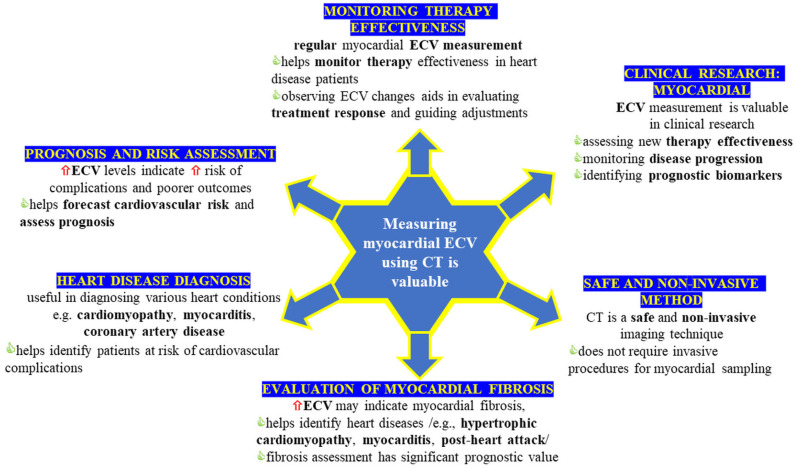
Determining the extracellular volume (ECV) of the myocardium through computed tomography (CT) provides significant value due to various factors. It illustrates how CT-derived ECV contributes diagnostically to identifying various cardiac pathologies, offers prognostic insights (for example, correlating with outcomes in heart failure and amyloidosis), and aids therapeutic decision-making, underscoring the importance of standardized imaging protocols and ongoing innovation. Red arrows (⇧) indicate an increase or elevation of the respective parameter.

Table 1 summarizes active clinical trials exploring ECV in cardiac conditions such as AF (NCT06308094), chronic kidney disease (NCT03704701), and oncological cardiotoxicity (NCT06048458). Figure 11 highlights ECV’s diagnostic, prognostic, and therapeutic potential, emphasizing the need for standardized protocols and ongoing innovation.

To enhance the practical applicability and clarity of this review, Table 5 summarizes specific cardiac diseases, emphasizing the clinical role of CT-derived ECV along with supporting evidence from studies included in our systematic review and meta-analysis.

### 4.7. Different Ranges of Radiation Dose

Radiation doses in CT-based ECV quantification vary significantly due to differences in scanner vendors, models, and imaging protocols. This variability stems from disparities in scanner technology, reconstruction algorithms, acquisition parameters, and protocol designs.

A total effective dose of 1.56 ± 0.58 mSv was reported for the DynEQ-CT protocol using the Siemens Somatom Sensation 64 scanner with prospective gating, emphasizing dose efficiency compared to alternative methods [9] Photon-counting detectors, such as Siemens NAEOTOM Alpha, achieved doses as low as 1.2 mSv, while traditional multiphase protocols exceeded 25 mSv [11]. DECT studies demonstrated doses ranging from 7.9 ± 3.1 mSv to 8.4 ± 2.4 mSv, with prospective ECG gating and iterative reconstruction playing key roles in minimizing exposure [19]. Lower doses (2.6 ± 0.9 mSv) were observed with a dual-layer spectral detector CT, attributed to standardized protocols and efficient scan designs [35].

Scanner type also influences dose variability. Dose-length product (DLP) ranged from 2093 to 5680 mGy·cm between Siemens models, highlighting the impact of scanner capabilities and acquisition settings [56]. Innovations like tin filtration and optimized energy settings enabled third-generation dual-source scanners to achieve lower doses [51].

Advancements in reconstruction algorithms and protocol standardization further impact dose efficiency. For instance, iterative reconstruction and optimized tube voltage settings (e.g., 90–120 kVp) have reduced radiation doses across studies.

Advances in reconstruction algorithms and optimized tube voltage (90–120 kVp) have further reduced doses across studies [37,41,54]. However, differences in dual-energy methodologies, such as rapid kVp switching versus dual-source systems, complicate direct comparisons [20,66].

The wide range of reported doses underscores the need for standardized protocols and advanced technologies, such as photon-counting detectors, to improve dose efficiency and ensure comparability across studies. Table 6 emphasizes the variability in radiation doses influenced by scanner type, vendor-specific technologies, and protocol optimizations.

## 5. Limitations, Strengths, and Future Directions

**Figure 12:**
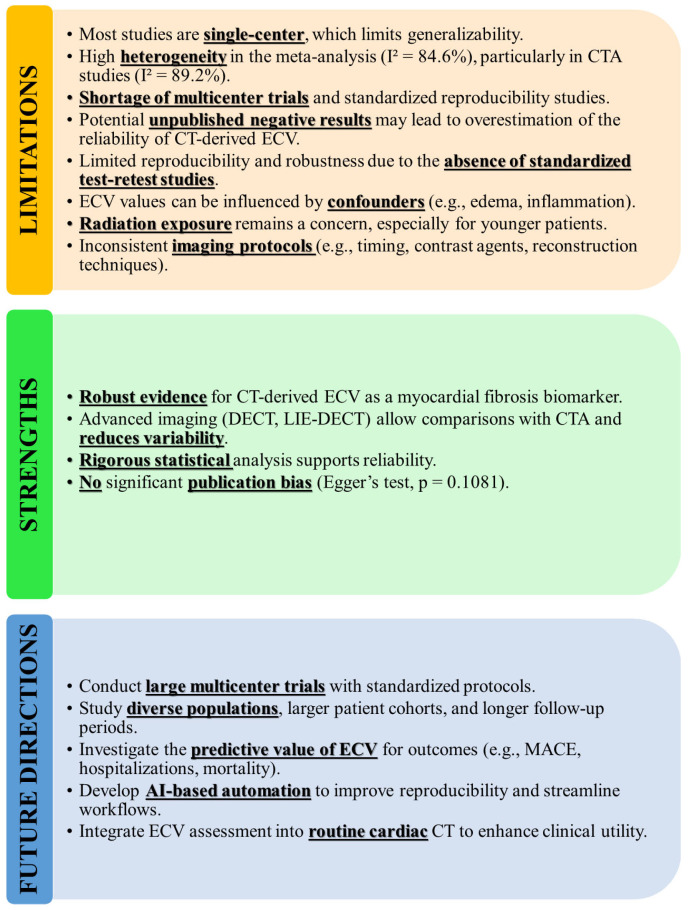
Summary of limitations, strengths, and future directions for CT-derived extracellular volume (ECV) quantification. This figure highlights key limitations, including high heterogeneity, limited multicenter trials, and confounding factors affecting reproducibility. Strengths include robust evidence for ECV as a myocardial fibrosis biomarker, advanced imaging techniques reducing variability, and rigorous statistical analysis with no significant publication bias. Future directions focus on conducting multicenter trials, studying diverse populations, exploring predictive value for outcomes, integrating AI-based automation, and routine implementation in cardiac CT workflows.

This review has several limitations which, along with its strengths and future directions, are presented in Figure 12.

Heterogeneity (I^2^ = 84.6%) reflects variability in imaging protocols, patient characteristics, and techniques. Egger’s test did not detect any significant publication bias; however, the absence of published negative studies could still lead to an overestimation of the reliability of CT-derived ECV. The performed meta-regression has certain limitations inherent to analyses based on aggregated data. Primarily, its statistical power may be restricted due to the limited number of studies included. In conditions like myocarditis or HF, elevated ECV may indicate edema, fibrosis, or fluid shifts rather than structural changes. Accurate diagnosis requires interpreting ECV alongside clinical, imaging, and laboratory data.

## 6. Conclusions

CT-derived myocardial ECV appears to be a promising biomarker for diagnosing and prognosticating cardiac conditions, particularly when MRI is unavailable or contraindicated, though further validation through multicenter studies and standardization of imaging protocols is essential before widespread clinical implementation. This meta-analysis highlights the potential clinical utility of advanced CT modalities such as DECT and LIE-DECT, although considerable variability due to differences in imaging protocols and patient populations underscores the need for standardization and caution in interpreting these findings. Specifically, clinical implementation is currently limited by important practical considerations, including patient exposure to ionizing radiation, potential inter-reader variability in ECV measurements, variations arising from different scanner technologies, and inconsistent contrast-administration protocols.

CT-based ECV is especially valuable in myocarditis, where elevated ECV correlates with heart dysfunction, arrhythmias, heart failure, and fibrosis. It aids in risk stratification and treatment planning for severe aortic stenosis, systemic amyloidosis, and anthracycline-induced cardiotoxicity. Additional uses include predicting outcomes in heart failure, tracking myocardial changes in chronic kidney disease, pulmonary hypertension, atrial fibrillation, and prognosticating MACE.

Technological advances, such as model-based iterative reconstruction and reduced contrast dosages, improve the accuracy and feasibility of CT-based ECV assessment. Compared to MRI, CT offers faster, more accessible evaluations.

Further research is needed to standardize CT protocols and validate findings across diverse populations to enhance clinical applicability. CT-derived ECV is a valuable, non-invasive biomarker for myocardial diagnosis, monitoring, and therapy.

## Figures and Tables

**Table 1:** Studies focused on ECV calculated from CT.

NCT Number (Acronym)	Title	Condition	Estimated Enrollment	Status	Location	Study Type
NCT06308094	320-detector Computed Tomography to Assess Myocardial Extracellular Volume Fraction in Patients With Atrial Fibrillation Before AF Ablation	Atrial Fibrillation	100	Not yet recruiting	Not provided	Interventional
NCT05717998	Imaging and Blood-Based Biomarkers for the Evaluation of Early Signs of Myocardial Injury After Thoracic Radiation Therapy	ECV calculated from MRI				
NCT04880317	Validation of Quantitative Myocardial Tissue Characterization Through Non-gated CT	Cardiac Disease; COVID-19; Cardiotoxicity; Extracellular Space Alteration	188	Recruiting	Italy	Interventional
NCT05877768 (EPIPHANY)	Evaluation of PCD-CT Based Image Parameters in the Assessment and Quantification of Coronary Artery Disease	Coronary Artery Disease	3000	Not yet recruiting	Germany	Observational
NCT06029400 (CTMyoC)	CT-based Myocardial Characterization (CTMyoC)	Different Cardiomyopathy and Major Cardiovascular Adverse Events	2200	Recruiting	Italy	Observational
NCT03704701 (TICKER)	The Interrogation of the Cardiomyopathy of Chronic Kidney Disease With advancEd caRdiac Imaging (TICKER)	Chronic Kidney Diseases; Cardiomyopathies; Cardiovascular Diseases; Cardiac Disease	28	Terminated	United Kingdom	Observational
NCT05326126	Microvascular Function in Patients Undergoing Transcatheter Aortic Valve Implant (TAVI) for Severe Symptomatic Aortic Stenosis: Association With Myocardial Fibrosis	Severe Symptomatic Aortic Stenosis	75	Recruiting	Italy	Interventional
NCT04392960	Novel Imaging Tools in Newly-diagnosed Patients With Cardiac AL Amyloidosis	ECV calculated from MRI				
NCT03029026 (ATTRact-AS)	The Role of Occult Cardiac Amyloid in the Elderly With Aortic Stenosis	Cardiac Amyloidosis; Aortic Stenosis	250	Unknown status	United Kingdom	Observational
NCT06020209 (CT-STEMI)	CCT for Comprehensive Risk Stratification Following STEMI	Myocardial Infarction	200	Recruiting	Italy	Interventional
NCT04625075 (MEMORY-COVID)	Manganese-Enhanced Magnetic Resonance Imaging of MyOcardial injuRY in COVID 19 (COVID-19) (MEMORY-COVID)	ECV calculated from MRI				
NCT05069168	Predictors Of Left Ventricular Systolic Function Recovery After Transcatheter Aortic Valve Replacement	Left Ventricular Systolic Function	30	Recruiting	Egypt	Observational
NCT05758493	Characterizing Iodine-124 Evuzumitide (AT-01) in Systemic Amyloidosis	ECV calculated mainly from MRI				
NCT06048458 (PC-TOX)	Cancer Treatment Related Cardiovascular Toxicity: Comprehensive Myocardial and Vascular Phenotyping	ECV calculated from MRI				
NCT02316587 (AMFAST)	Assessment of Myocardial Fibrosis in Aortic STenosis (AMFAST)	Endomyocardial Fibrosis; Aortic Valve Stenosis	112	Completed	Denmark	Observational
NCT05479669 (VIP-HF2)	Value of Intense Phenotyping in Heart Failure With Preserved Ejection Fraction	ECV calculated from MRI				

**Table 4:** Characteristics of the studies included in the meta-analysis evaluating extracellular volume (ECV) measurement using computed tomography (CT). Studies are divided based on imaging methods: advanced imaging techniques (DECT, LIE-DECT) and standard CT-based techniques (CTA). The differences in 95% confidence intervals (CI) between the input data table and the forest plot arise from the application of random-effects modeling in R, which incorporates both within-study variance and between-study heterogeneity. This approach often results in wider CIs in the forest plot compared to the original study-level data, reflecting the additional uncertainty due to variability in study populations, methodologies, or imaging protocols. These discrepancies underscore the importance of selecting an appropriate statistical model when interpreting aggregated results in meta-analyses.

Authors (Year) [Ref.]	ECV Measurement Method	n_1_	n_2_	%ECV_1_ [Mean (SD)]	%ECV_2_ [Mean (SD)]	Group 1 (Higher ECV or Pathological Condition)	Group 2 (Lower ECV or Control)	Effect Size (SMD)	95% CI (Lower, Upper)	SE	1/SE (Precision)	ICC
(1) Chevance et al. (2018) [19]	DECT	22	13	56.0 (7.0)	36.0 (5.0)	CA	CH	3.15	(2.14, 4.16)	0.51	1.95	NA
(2) Ohta et al. (2019) [20]	DECT	11	35	31.35 (2.53)	26.63 (2.69)	Non-ischemic DCM	Healthy control	1.78	(1.01, 2.55)	0.39	2.55	NA
(3) Si-Mohamed et al. (2021) [21]	DECT	60	12	34.18 (3.33)	30.04 (2.25)	Myocarditis	Healthy control	1.30	(0.64, 1.96)	0.33	2.99	NA
(4) Qi et al. (2022) [22]	LIE-DECT	77	80	31.5 (4.3)	26.8 (2.8)	Non-ischemic HFpEF	Healthy control	1.30	(0.96, 1.64)	0.18	5.69	NA
(5) Qi et al. (2022) [22]	LIE-DECT	35		31.7 (3.7)		Ischemic HFpEF		1.58	(1.13, 2.03)	0.23	4.39	
(6) Qi R-X et al. (2021) [23]	LIE-DECT	60	60	31.3 (4.0)	27.1 (3.7)	HF without CAD	Healthy control	1.09	(0.71, 1.47)	0.20	5.11	NA
(7) Han et al. (2021) [24]	CTA	70	39	33.2 (7.7)	29.4 (6.1)	AS with no LVEF improvement post-TAVR	AS with LVEF improvement post-TAVR	0.53	(0.13, 0.93)	0.20	4.93	0.91
(8) Tamarappoo et al. (2020) [25]	CTA	57	93	40.1 (6.0)	25.6 (4.6)	Patients with ECV > 33%, higher risk of death and HF hospitalization	Patients with ECV ≤ 33%, lower risk of death and HF hospitalization	2.80	(2.34, 3.26)	0.23	4.28	0.94
(9) Gama et al. (2022) [26]	CTA	37	35	56.0 (11.0)	43.0 (13.0)	ATTR	AL	1.08	(0.59, 1.58)	0.25	3.96	NA
(10) Hammer et al. (2021) [27]	CTA	75	19	40.0 (11.0)	21.6 (5.6)	AS	Healthy control	1.81	(1.24, 2.37)	0.29	3.46	0.93
(11) Hamdy et al. (2019) [28]	CTA	35	17	39.6 (5.3)	27.1 (2.1)	MI	Healthy control	2.76	(1.97, 3.55)	0.40	2.50	NA
(12) Yashima et al. (2023) [29]	CTA	21	49	37.16 (5.91)	32.59 (3.95)	DCM with MACE	DCM without MACE	0.99	(0.45, 1.53)	0.27	3.65	NA
(13) Yamada et al. (2020) [30]	CTA	20	20	33.8 (4.7)	26.6 (2.9)	HD	Healthy control	1.84	(1.10, 2.58)	0.38	2.65	0.96
(14) Nishikawa et al. (2023) [31]	CTA	9	24	39 (5)	33 (5)	After CatAbl due to AF without RR	After CatAbl due to AF with RR	1.20	(0.38, 2.02)	0.42	2.39	NA
(15) Egashira et al. (2021) [32]	CTA	7	37	30.3 (4.8)	26.2 (2.5)	CTRCD (anthracycline)	After anthracycline therapy without CTRCD	1.39	(0.54, 2.25)	0.44	2.28	NA

Abbreviations: 95%CI, 95% confidence interval; AF, atrial fibrillation; AL, light-chain amyloidosis; AS, aortic stenosis; ATTR, transthyretin amyloidosis; CAD, coronary artery disease; CA, cardiac amyloidosis; CatAbl, catheter ablation; CH, Cardiac Hypertrophy; CTA, computed tomography angiography; CTRCD, Cancer Therapeutics-Related Cardiac Dysfunction; DCM, dilated cardiomyopathy; DECT, dual-energy computed tomography; ECV, extracellular volume; HD, hemodialyzed; HF, heart failure; HFpEF, heart failure with preserved ejection fraction; ICC, Intraclass Correlation Coefficient; LIE-DECT, late iodine enhancement dual-energy computed tomography; LVEF, left ventricular ejection fraction; MACE, major adverse cardiac events; MI, myocardial infarction; NA, Not Applicable; RR, reverse remodeling; SD, standard deviation; SE, standard error; SMD, standardized mean difference; TAVR, transcatheter aortic valve replacement.

**Table 5:** Clinical role and evidence for CT-derived extracellular volume (ECV) in specific cardiac diseases.

Disease	Clinical Role of CT-Derived ECV	Supporting Evidence
Myocarditis	Diagnostic (inflammation, fibrosis detection); prognostic (risk stratification)	Elevated ECV significantly distinguishes myocarditis patients from controls (e.g., 34.18% vs. 30.04%), aiding diagnosis and prognosis [21]
Cardiac Amyloidosis	Diagnostic (amyloid burden quantification); prognostic (severity, mortality)	Consistently elevated ECV (>40–50%) strongly correlates with myocardial amyloid deposition, disease severity, and increased mortality risk [19,26]
Dilated Cardiomyopathy (DCM)	Prognostic (predicting adverse cardiac events)	Higher ECV (>32%) associated with increased risk of major adverse cardiac events and worse clinical outcomes [29]
Heart Failure with Preserved EF (HFpEF)	Prognostic (cardiac remodeling, risk stratification)	Increased ECV (>31%) associated with higher NT-proBNP, worse NYHA class, and adverse outcomes, predicting HF progression [22]
Aortic Stenosis (AS)	Prognostic (post-TAVI outcomes, myocardial fibrosis severity)	High pre-TAVI ECV (>33%) strongly predicts poor clinical outcomes, reduced LV functional recovery, and increased HF hospitalizations [24,27]
Anthracycline-induced Cardiotoxicity	Diagnostic and monitoring (early detection of myocardial injury)	Elevated ECV post-anthracycline (e.g., >30%) identifies early myocardial injury, preceding LVEF reduction, enabling early intervention [32]
Chronic Kidney Disease (CKD)	Monitoring (cardiovascular remodeling, fibrosis)	ECV elevation correlates with LV remodeling, higher cardiovascular risk, and fibrosis extent in CKD patients undergoing dialysis [30]
Pulmonary Hypertension (PH)	Monitoring and prognostic (severity, response to therapy)	ECV correlates positively with pulmonary artery pressure and right ventricular remodeling, effectively monitoring treatment response and disease severity [70,72]
Atrial Fibrillation (AF)	Prognostic (reverse remodeling prediction post-ablation)	Patients with lower baseline ECV have significantly better reverse remodeling outcomes post-ablation [31]

Abbreviations: CT, computed tomography; ECV, extracellular volume; LV, left ventricle; EF, ejection fraction; NYHA, New York Heart Association; NT-proBNP, N-terminal pro-brain natriuretic peptide; TAVI, transcatheter aortic valve implantation.

**Table 6:** Summary of radiation doses by vendor, scanner, and protocol.

Authors [Ref.]	Scanner Model	Vendor	Protocol	Effective Dose (mSv)	Notes
Treibel et al. [9]	Somatom Sensation 64	Siemens Healthineers	DynEQ-CT	1.56 ± 0.58	Prospective gating; chest-specific conversion factor.
Cundari et al. [11]	Various	Multiple	Various	1.2–25	Photon-counting detectors significantly lower doses.
Chevance et al. [19]	DECT (model not specified)	Not specified	DECT	7.9 ± 3.1 to 8.4 ± 2.4	Prospective ECG triggering; ASIR technology.
Liu et al. [35]	iQon Spectral CT	Philips Healthcare	DECT	2.6 ± 0.9	Standardized dual-layer spectral detector CT protocol.
Monti et al. [56]	Somatom Definition/Emotion 16	Siemens Healthineers	Body CT	DLP 2093–5680 mGy·cm	Significant variability due to scanner capabilities and settings.
Abadia et al. [51]	Somatom Definition Flash/Force	Siemens Healthineers	DECT	Not reported	Dose reductions with tin filtration and advanced dual-energy features.

Abbreviations: ASIR, Adaptive Statistical Iterative Reconstruction; CT, computed tomography; DECT, dual-energy computed tomography; DynEQ-CT, dynamic equilibrium computed tomography; ECG, Electrocardiogram.

## Data Availability

The original contributions presented in this study are included in the article. Further inquiries can be directed to the corresponding author.

## Abbreviations

The following abbreviations are used in this manuscript:

AF	Atrial Fibrillation
AHA	American Heart Association
AL	Amyloidosis
AS	Aortic Stenosis
ATTR	Amyloid Transthyretin
AUC	Area Under The Curve
BNP	B-Type Natriuretic Peptide
CA	Cardiac Amyloidosis
CCT	Cardiac Computed Tomography
CCTA	Coronary Computed Tomography Angiography
CI	Confidence Interval
CTEPH	Chronic Thromboembolic Pulmonary Hypertension
CTRCD	Cancer Therapy-Related Cardiac Dysfunction
DCM	Dilated Cardiomyopathy
DE	Dual Energy
DECT	Dual-Energy CT
DESCT	Delayed-Enhancement Spectral Computed Tomography
DLCT	Dual-Layer Computed Tomography
DLP	Dose Length Product
ECV	Extracellular Volume
ECViodine	Extracellular Volume Calculated Using Iodine Contrast Agent
ECVsub	Extracellular Volume Of A Specific Myocardial Subregion
FFR	Fractional Flow Reserve
GLS	Global Longitudinal Strain
HFpEF	Heart Failure With Preserved Ejection Fraction
HR	Hazard Ratio
HU	Hounsfield Units
ICC	Intraclass Correlation Coefficient
id-CT	Iodine Density-Derived CT
IVST	Interventricular Septal Thickness
LAV	Left Atrial Volume
LAVI	Left Atrial Volume Index
LE	Late Enhancement
LE-CT	Late Enhancement CT
LGE	Late Gadolinium Enhancement
LIE-DECT	Late Iodine Enhancement Dual-Energy Computed Tomography
LV	Left Ventricle
LVEDV	Left Ventricular End-Diastolic Volume
LVEDVI	Left Ventricular End-Diastolic Volume Index
LVEF	Left Ventricular Ejection Fraction
LVESV	Left Ventricular End-Systolic Volume
LVESVI	Left Ventricular End-Systolic Volume Index
LVID	Left Ventricular Internal Diameter
LVM	Left Ventricular Mass
LVMM	Left Ventricular Myocardial Mass
LVMMI	Left Ventricular Myocardial Mass Index
MACE	Major Adverse Cardiovascular Events
MIC	Myocardial Iodine Concentration
MIF	Myocardial Interstitial Fibrosis
mPAP	Mean Pulmonary Artery Pressure
NACH	Non-Amyloid Hypertrophic Heart
NOS	Newcastle–Ottawa Scale
NYHA	New York Heart Association
PCD-CT	Photon-Counting Detector Computed Tomography
PCI	Percutaneous Coronary Intervention
PH	Pulmonary Hypertension
PWT	Posterior Wall Thickness
ROC	Receiver Operating Characteristic
RVIP	Right Ventricular Insertion Point
SAVR	Surgical Aortic Valve Replacement
SDCT	Spectral Dual Detector CT
sd-CT	Subtraction-Derived CT
SE	Standard Error
SECT	Single-Energy CT
SMD	Standard Mean Deviation
SSDE-CT	Single Source Dual-Energy CT
TAVI	Transcatheter Aortic Valve Implantation
TAVR	Transcatheter Aortic Valve Replacement
TNF	Tumor Necrosis Factors
TRO	Triple-Rule-Out
VUE	Virtual Unenhanced Attenuation
